# Hepatoprotective Triterpene Saponins from the Roots of *Glycyrrhiza inflata*

**DOI:** 10.3390/molecules20046273

**Published:** 2015-04-09

**Authors:** Yun-Feng Zheng, Juan-Hua Wei, Shi-Qi Fang, Yu-Ping Tang, Hai-Bo Cheng, Tian-Lin Wang, Cun-Yu Li, Guo-Ping Peng

**Affiliations:** 1 School of Pharmacy, Nanjing University of Chinese Medicine, Nanjing 210023, China; E-Mails: zyunfeng88@126.com (Y.-F.Z.); weijuanhua1989@163.com (J.-H.W.); fangsq8866@163.com (S.-Q.F.); yupingtang9@126.com (Y.-P.T.); cpuwtl@gmail.com (T.-L.W.); licunyuok@163.com (C.-Y.L.); 2 Jiangsu Collaborative Innovation Center of Chinese Medicinal Resources Industrialization, Nanjing 210023, China; 3 Translational Medicine Research Center of Nanjing University of Chinese Medicine, Nanjing 210023, China; E-Mail: nzychb@163.com

**Keywords:** *Glycyrrhiza inflate*, genus *Glycyrrhiza*, triterpene saponin, hepatoprotective, phospholipase A2

## Abstract

Two novel oleanane-type triterpene saponins, licorice-saponin P2 (**1**) and licorice-saponin Q2 (**3**), together with nine known compounds **2**, **4**–**11**, have been isolated from the water extract of the roots of *Glycyrrhiza inflata*. The structures of these compounds were elucidated on the basis of spectroscopic analysis, including 2D-NMR experiments (^1^H–^1^H COSY, HSQC, HMBC and ROESY). In *in vitro* assays, compounds **2**–**4**, **6** and **11** showed significant hepatoprotective activities by lowering the ALT and AST levels in primary rat hepatocytes injured by D-galactosamine (D-GalN). In addition, compounds **2**–**4**, **6**, **7** and **11** were found to inhibit the activity of PLA_2_ with IC_50_ values of 6.9 μM, 3.6 μM, 16.9 μM, 27.1 μM, 32.2 μM and 9.3 μM, respectively, which might be involved in the regulation of the hepatoprotective activities observed.

## 1. Introduction

The genus *Glycyrrhiza* consists of about 30 species with a nearly global distribution, of which 18 species are found in China. Among them, three species named *Glycyrrhiza uralensis*, *Glycyrrhiza glabra* and *Glycyrrhiza inflata*, have been used as traditional Chinese medicine for the treatment of hepatitis, spasmodic cough, gastric ulcer, and so on. Phytochemical studies have showed that triterpenoid saponins and flavonoids were the two of major kinds of active substances of *Glycyrrhiza*, which have a variety of pharmacological activities, including hepatoprotective [1,2], antiviral [3], anti-inflammatory [4] and antioxidative [5] effects. Recently, we reported the chemical constituents of *G. uralensis* and *G. glabra*, as well as their cytotoxic or neuraminidase bioactivities [6,7]. As part of our ongoing research on the genus *Glycyrrhiza*, an extensive phytochemical investigation on the roots of *G. inflata* has now led to the isolation of two new oleanane-type saponins **1**, **3** and nine known saponins **2**, **4–11**. All compounds were screened for their protective activities against D-galactosamine (D-GlaN) induced toxicity *in vitro*. In addition, the inhibitory activities on phospholipase A2 (PLA2) were presented. Herein, we report the isolation and structural elucidation of these saponins, along with the investigation of their protective activities.

## 2. Results and Discussion

**Figure 1:**
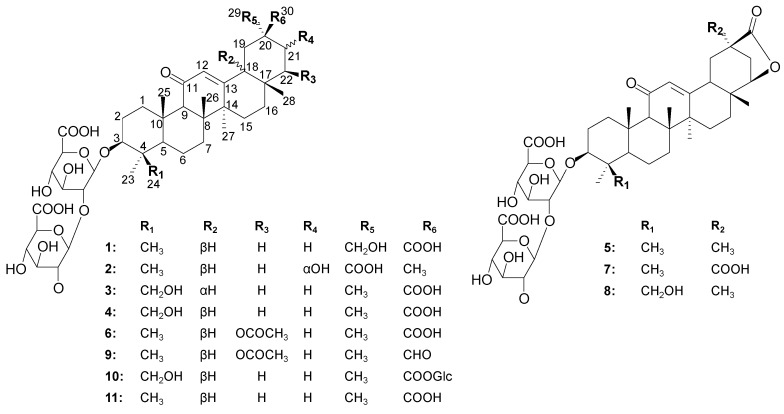
Chemical structures of compounds **1–11**.

The total saponin fraction of *G. inflata* was prepared by co-application of polyamide and macroporous resin column chromatography [7]. The resulting extract was subjected to ODS column chromatography and preparative HPLC to afford two new oleanane-type saponins **1, 3** together with nine known ones **2**, **4–11**. Their structures were shown in Figure 1. 

### 2.1. Structural Determination

Compound **1** was obtained as a white amorphous powder and showed a protonated peaks in the low-resolution positive HR-ESI-MS spectrum at *m/z* 861.3929 [M + Na]^+^ and *m/z* 839.4120 [M + H]^+^. Its molecular formula was thus determined as C_42_H_62_O_17_, requiring 12 degrees of unsaturation. The UV spectrum showed an absorption maximum at 250.8 nm (MeOH, log ε 4.14), indicating the presence of an α,β-unsaturated carbonyl moiety. In the ^1^H-NMR and ^13^C-NMR spectra, representative signals of eight tertiary carbons at δ_C_ 75.3–85.5, two carboxyl carbons at δ_C_ 174.8 and 174.8, and two anomeric carbons δ_C_ 105.9, δ_H_ 5.03 (1H, d, *J* = 8.0 Hz) and 107.3, 5.38 (1H, d, *J* = 7.5 Hz) suggested the presence of two monosaccharide residues. The coupling constants of the anomeric protons indicated glycosidic bonds with β-configurations [8]. Acid hydrolysis and GC comparison with authentic samples indicated the presence of D-glucuronic acid (GlcA) [9]. This deduction was further supported by the signals of two fragment ion peaks at *m*/*z* 663.3762 [M + H − C_6_H_8_O_6_]^+^ and *m*/*z* 487.3448 [M + H − 2C_6_H_8_O_6_]^+^ in the HR-ESI-MS spectrum. The glycosidic site was established unambiguously by a HMBC experiment in which a long-range correlation between H-1*′* (δ_H_ 5.03) and C-3 (δ_C_ 91.3), H-1*′′* (δ_H_ 5.38) and C-2*′* (δ_C_ 85.5). Thus, the carbohydrate sequence of **1** was established as 3-*O*-β-d-glucuronopyranosyl-(1→2)-β-d-glucuronopyranosyl. The ^1^H-NMR spectrum of the aglycone moiety of **1** showed signals corresponding to six tertiary methyls [δ_H_ 1.27, 1.08, 1.07, 0.85, 1.36 and 0.70, (each 3H, s)], one oxygenated methylene [δ_H_ 4.06, 3.98, (2H, d, 10.5)], one oxygenated methine [δ_H_ 3.27 (1H, dd, 4.0 and 11.5)], and one unsaturated methine [δ_H_ 5.83 (1H, s)], while the ^13^C NMR and DEPT spectrum displayed 30 carbon resonances, containing six methyls, ten methylenes (including one oxygenated methylene), five methines (including one oxygenated methine and one unsaturated methine), and nine quaternary carbons (including one carbonyl quaternary carbon, one unsaturated quaternary carbon and one carboxyl carbon). Therefore, compound **1** was considered to be an oleanane-type triterpene glucuronide bearing a 12(13)-double bond and a keto group at C-11. In the HMBC spectrum, correlations of δ_H_ 5.03 (H-1*′*) to δ_C_ 91.3 (C-3) and δ_H_ 5.38 (H-1*′′*) to δ_C_ 85.5 (C-2*′*) could be observed. In addition, the correlations in the HMBC spectrum from H-1*′* at δ_H_ 5.03, H-23 at δ_H_ 1.27 and H-24 at δ_H_ 1.08 to C-3 at δ_C_ 91.3 helped in assigning one oxygenated methine at C-3. 

**Figure 2:**
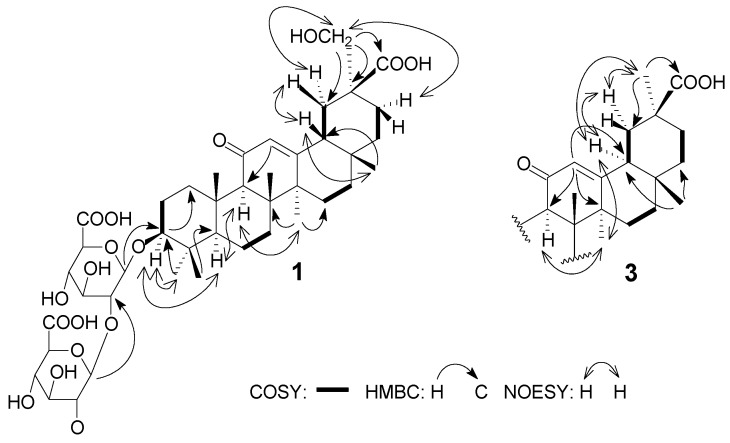
Key COSY, NOESY and HMBC correlations of compounds **1** and **3**.

Detailed analysis of the above 1D-NMR data and 2D-NMR correlations indicated that **1** is an oleanane-type saponin derivative and is structurally related to the known compound licorice-saponin G2 (**4**). The comparison of the NMR data of **1** with those of **4** suggested that the hydroxyl group at C-24 in **4** was transposed to C-29 in **1**. The HMBC correlations from δ_H_ 3.98, 4.06 (H-29) to δ_C_ 39.1 (C-19) and δ_C_ 180.2 (C-30) and the ^1^H–^1^H COSY correlations between the proton signal at δ_H_ 2.49 (H-18) and δ_H_ 1.98, 2.24 (H-19) confirmed that hydroxyl group was connected to C-29 in compound **1** (Figure 2).

The relative configuration of **1** was established on the basis of NOESY correlations of H-3 with H-1'α, H-23 and H-5, H-9 with H-5 and H-27, and H-18 with H-28β and H-19β, as well as H-29 with H-19α, H-21α which revealed that the substituent groups of C-3, H-18 were β-oriented and C-29 was α-oriented. From these spectroscopic data, compound **1** was deduced to be 3β-*O*-[β-d-glucuronopyranosyl-(1→2)-β-d-glucuronopyranosyl]-29-hydroxyglycyrrhetic acid, and named licorice-saponin P2. The structure of compound **1** was confirmed to be as shown in Figure 1 and the ^1^H- and ^13^C-NMR data are listed in Table 1.

**Table 1:** NMR Chemical Shifts of Compounds **1–3** (C_5_D_5_N, 500 MHz).

Position	1		2		3	
	δC mult	δH (J in Hz)	δC mult	δH (J in Hz)	δC mult	δH (J in Hz)
1	41.4 CH2	0.89 *,2.81 (m)	40.5 CH2	1.05 *,3.00 (m)	40.3 CH2	1.03 *,2.97(m)
2	28.6 CH2	1.84 (m),2.11 (m)	27.7 CH2	1.98 (m),2.26 (m)	27.3 CH2	2.12(m),2.23(m)
3	91.3 CH	3.27 (dd,4.0,11.5)	90.3 CH	3.37(dd,4.0,11.5 )	90.4 CH	3.53(dd,4.5,11.5)
4	41.8 qC		40.9 qC		45.2 qC	
5	57.3 CH	0.62 (m)	56.4 CH	0.71 (m)	56.7 CH	0.87 *
6	19.5 CH2	1.18 *,1.41 (m)	18.6 CH2	1.25 *,1.49 (m)	19.1 CH2	1.55 *,1.72 *
7	34.8 CH2	1.12 *,1.47 (m)	34.1 CH2	1.22 *,1.49 (m)	33.9 CH2	1.28 *,1.54 *
8	47.6 qC		46.5 qC		46.4 qC	
9	64.0 CH	2.31 (s)	63.1 CH	2.44 (s)	62.7 CH	2.43(s)
10	34.6 qC		38.3 qC		37.8 qC	
11	202.4 qC		201.1 qC		200.5 qC	
12	130.3 CH	5.83 (s)	129.7 CH	5.89 (s)	129.4 CH	5.82(s)
13	173.1 qC		171.7 qC		170.6 qC	
14	45.5 qC		45.0 qC		44.4 qC	
15	28.6 CH2	1.04 *,1.64 (m)	27.9 CH2	1.05 *,1.68 (m)	27.4 CH2	1.08 *,1.70 (m)
16	28.6 CH2	0.90 *,2.11 (m)	31.1 CH2	1.28 *,3.09 (m)	27.3 CH2	0.95 *,2.10 (m)
17	34.6 qC		34.1 qC		33.3 qC	
18	50.3 CH	2.49 (m)	48.2 CH	2.37 (m)	47.5 CH	2.26 (m)
19	39.1 CH2	1.98 *,2.24 (m)	36.1 CH2	1.68 *,3.13 (m)	40.6 CH2	1.59 *,2.47(m)
20	53.0 qC		49.1 qC		43.4 qC	
21	28.6 CH2	1.99 *,2.23 (m)	73.1 CH	4.52 (m)	30.5 CH2	1.68 *,2.18(m)
22	39.8 CH2	1.42 *,1.61 (m)	44.0 CH2	1.78 *,1.98 (m)	36.6 CH2	1.38 *,1.53 *
23	30.1 CH3	1.27 (s)	29.1 CH3	1.35 (s)	23.7 CH3	1.49 (s)
24	18.9 CH3	1.08 (s)	18.0 CH3	1.2 (s)	64.2 CH2	3.72 (d, 12),4.40*
25	18.8 CH3	1.07 (s)	17.8 CH3	1.21 (s)	17.4 CH3	1.21 (s)
26	20.8 CH3	0.85 (s)	19.9 CH3	1.08 (s)	19.4 CH3	1.07 (s)
27	25.6 CH3	1.36 (s)	24.1 CH3	1.46 (s)	24.3 CH3	1.40 (s)
28	30.8 CH3	0.70 (s)	30.2 CH3	0.93 (s)	29.3 CH3	0.88 (s)
29	72.9 CH2	4.06 (d, 10.5), 3.98 (d, 10.5)	181.0 qC		20.6 CH3	1.42 (s)
30	180.2 qC		22.0 CH3	1.44 (s)	181.7 qC	
1'	106.7 CH	5.03 (d, 8.0)	105.9 CH	5.09 (d, 7.5)	105.3 CH	5.09 (d, 8.0)
2'	85.5 CH	4.18 *	84.8 CH	4.26 *	81.6 CH	4.38 *
3'	79.4 CH	4.49 *	78.6 CH	4.54 *	78.6 CH	4.56 *
4'	75.3 CH	4.38 *	78.2 CH	4.69 *	78.3 CH	4.71 *
5'	79.3 CH	4.65 *	74.2 CH	4.53 *	74.1 CH	4.55 *
6'	174.8 qC		173.8 qC		173.4 qC	
1''	108.1 CH	5.38 (d, 7.5)	107.3 CH	5.45(d, 7.5)	105.5 CH	5.77 (d, 8.0)
2''	78.5 CH	4.14 *	77.6 CH	4.22 *	76.5 CH	4.31 *
3''	79.4 CH	4.32 *	78.5 CH	4.42 *	78.5 CH	4.42 *
4''	75.3 CH	4.47 *	74.4 CH	4.62 *	78.4 CH	4.63 *
5''	80.1 CH	4.51 *	79.2 CH	4.61 *	73.9 CH	4.62 *
6''	174.8 qC		173.4 qC		173.2 qC	

* where ^1^H-NMR signals were overlapped, chemical shift data were obtained from 2D correlations.

Compound **3** produced a protonated ion at *m*/*z* 839.4069 [M + H]^+^ by HR-ESI-MS, which indicated its molecular formula C_42_H_62_O_17_. In the ^13^C-NMR spectrum eight tertiary carbons at δ_C_ 70–85, two carboxyl carbons at δ_C_ 173.4 and 173.2, and two anomeric carbons δ_C_ 105.3 and 105.5 suggested the presence of two glucuronopyranosyl moieties. This conclusion was confirmed by two fragment ion peaks at *m*/*z* 663.3785 [M − C_6_H_8_O_6_ + H]^+^ and 487.3373 [M − 2C_6_H_8_O_6_ + H]^+^, as well as acid hydrolysis and GC analysis. 

The NMR spectrum of **3** were very similar to that of **4**, and detailed analysis revealed that the main difference could be seen in the NOESY spectrum. Comparative analysis of this spectrum showed that correlations of H-18 with H-19α and H-29 in **3** (Figure 2), rather than correlations of H-18 with H-28β in **4**, were present, which indicated that H-18 of **3** was α-oriented. The other correlations of **3**, including H-1β/H-25 and H-26, H-3/H-5 and H-1α, H-23/H-3 and H-1', were the same as those of **4**. Accordingly, compound **3** was identified as 3β-*O*-[β-D-glucuronpyranosyl-(1→2)-β-D-glucuron-pyranosyl]-24-hydroxy-18α-glycyrrhetic acid, which was assigned the trivial name licorice-saponin Q2.

The known constituents, namely macedonoside A (**2**) [10], licorice-saponin G2 (**4**) [11], licorice-saponin E2 (**5**) [12], 22β-acetoxyglycyrrhizin (**6**) [13], uralsaponin D (7) [7], 24-hydroxylicorice-saponin E2 (**8**) [14], 22β-acetoxyglycyrrhaldehyde (**9**) [15], licorice-saponin A3 (**10**) [12] and glycyrrhizin (**11**) [15] were identified by comparison of their NMR data with the literature data. 

### 2.2. Hepatoprotective Activity 

All the separated compounds were assessed for their hepatoprotective activities against the increase of AST and LDH levels in primary rat hepatocytes injured by d-GalN. The maximum nontoxic concentrations of tested compounds on primary rat hepatocytes were in the range of 120–240 μM. A set of cells in culture medium treated with d-GalN was used as the model group, and in comparison to the model group, macedonoside A (**2**), licorice-saponin Q2 (**3**), licorice-saponin G2 (**4**), 22β-acetoxy-glycyrrhizin (**6**) and glycyrrhizin (**11**) notably lowered AST (10.3–16.5 U·L^−1^) and LDH (200.7–242.8 U·L^−1^) in the range of concentration 30–120 μM. (Table 2).

**Table 2:** Hepatoprotective activities of isolated saponins on AST and LDH Levels in primary cultures of rat hepatocytes injured by d-GalN ^a^.

Groups	Concentration (μM)	AST (U·L^−1^)	LDH (U·L^−1^)
Control		6.9 ± 1.7	157.4 ± 11.7
Model		17.1 ± 2.4 ^b^	253.5 ± 13.5 ^b^
2	30	14.6 ± 3.1	231.0 ± 18.3 ^c^
	60	13.1 ± 2.1 ^c^	213.7 ± 19.6 ^d^
	120	11.2 ± 1.9 ^d^	215.9 ± 8.2 ^d^
3	30	13.3 ± 2.9 ^c^	224.6 ± 21.4 ^c^
	60	12.9 ± 3.2 ^d^	208.6 ± 16.0 ^d^
	120	10.8 ± 2.8 ^d^	200.7 ± 15.1 ^d^
4	30	14.8 ± 2.9	237.2 ± 16.4
	60	13.7 ± 2.6 ^c^	232.7 ± 25.7
	120	12.6 ± 2.8 ^c^	224.9 ± 25.2 ^c^
6	30	16.5 ± 2.2	242.8 ± 15.2
	60	14.6 ± 3.0	228.7 ± 14.0 ^c^
	120	13.2 ± 2.7 ^c^	221.7 ± 19.5 ^d^
11	30	14.1 ± 2.1 ^c^	236.3 ± 9.4 ^c^
	60	13.0 ± 2.2 ^c^	219.1 ± 19.3 ^d^
	120	11.6 ± 1.8 ^d^	207.8 ± 21.9 ^d^
Silibinin Meglumine	50	10.3 ± 2.2 ^d^	219.1 ± 10.9 ^d^

^a^ *n* = 3, mean ± SD. Control: a set of hepatocytes maintained in culture medium. Model: a set of hepatocytes maintained in culture medium and treated only with D-GalN. ^b^ *p* < 0.01, compared to control group. ^c^ *p* < 0.05, compared to model group. ^d^ *p* < 0.01, compared to model group.

Comparing the activities of these saponins, compound **5** and **7** was shown to have significantly weaker hepatoprotective activities than the compound **2** and **11** owing to presence of a lactone ring at position 22(30). Compound **11** showed stronger activity than **1**. That might be because an additional CH_2_OH group is preferable to improve the steric hindrance, thus resulting in a decrease in the bonding capacity with active targets. Interestingly, compound **3** displayed higher activity than compound **4**. The reason might be that compound **3** with a 18α-H group was found to be favorable for the anti-liver injury activity. On the basis of the above analysis, it seemed that a carboxyl residue at position 29 or 30 was possibly the necessary group for hepatoprotective activity.

### 2.3. Enzyme Inhibition Activity

As a regulator associated with the stability of the liver cell membrane, phospholipase A2 (PLA_2_) is a promising target for hepatoprotective drug development [16]. To examine whether the compounds inhibit activities on PLA_2_, the enzyme inhibitory potency of all isolated compounds was conducted and the results were summarized in Table 3. Among these, two saponins (compounds **2** and **3**) and glycyrrhizin (**11**) exhibited efficient inhibitory activity with IC_50_ value of 6.9 μM, 3.6 μM and 9.3 μM, respectively. Compounds **4**, **6** and **7** showed moderate inhibitory activities with IC_50_ values of 16.9 μM, 27.1 μM and 32.2 μM, respectively. 

What was noteworthy, is that analysis of the two assays of **1–11** showed that there was good relationship between PLA_2_ inhibitory activities and hepatoprotective effects, leading to the hypothesis that inhibition of PLA_2_ was one of the possible mechanisms of the hepatoprotective effect of licorice saponins.

**Table 3:** Inhibitory activities of isolated saponins on PLA_2_.

Compounds	IC_50_ (μM)
**1**	>50
**2**	6.9 ± 0.5
**3**	3.6 ± 0.3
**4**	16.9 ± 0.3
**5**	>50
**6**	27.1 ± 0.9
**7**	32.2 ± 0.5
**8**	>50
**9**	>50
**10**	>50
**11**	9.3 ± 0.8
Diethylenetriaminepentaacetic acid	1.8 ± 0.1

## 3. Experimental Section

### 3.1. General Procedures

UV-vis spectra were recorded using a UV-2401 spectrophotometer (Shimadzu, Kyoto, Japan). The 1D and 2D-NMR spectra (^1^H-^1^H COSY, NOESY, HSQC and HMBC) were obtained using an ASR-500 spectrometer (Bruker, Fällanden, Switzerland, 500 MHz for ^1^H and 500 MHz for ^13^C spectra). All compounds were dissolved in C_5_D_5_N and chemical shifts were reported in ppm (δ) relative to TMS. HR-TOF-MS was recorded on a Bruker MicroTOF-Q spectrometer. Column chromatography was performed on polyamide resin (100–200 mesh) and macroporous resin (20–40 mesh, AB-8) columns. Medium pressure liquid chromatography (MPLC) was carried out on a LISURE apparatus (Chromatography Pump E2-purifier, Lisure Science Co., Ltd, Suzhou, China) with ODS column (6.5 cm × 50 cm; 25–50 μm, Merck K GaA). Preparative HPLC was performed by Econosil C18 column (22 × 250 mm; 10 μm, Alltech, Lexington, KY, USA) on a Waters 600 HPLC instrument (Waters, Milford, MA, USA). HPLC was performed on an Agilent 1100 HPLC instrument (Agilent Technologies Inc., Palo Alto, CA, USA) connected to a UV detector, which was equipped with a Thermo C_18_ column (250 mm × 4.6 mm, I.D. 5 μm). GC was run on Varian CP-3800 Gas Chromatograph (VARIAN, INC., Palo Alto, CA, USA) equipped with a CP-sil 5 CB capillary column (30 m, 0.25 mm i.d., 0.25 μm) and a Saturn 2200 Mass detector. The chemical reagents were supplied by Nanjing Chemical Plant (Nanjing, China). The fluorescence value of each well in the biological activity assays was read in a ELISA plate reader (Bio-Tek Instruments, Winooski, VT, USA). 

### 3.2. Material

The roots of *Glycyrrhiza inflata* were collected in Weli County, Xinjiang Uygur Autonomous Region, China, October 2013. A voucher sample (No. 20131015) was preserved in Nanjing University of Chinese Medicine, and identified by Prof. Qi-Nan Wu.

### 3.3. Extraction and Isolation 

The roots of *G. inflata* (dry weight, 25 kg) were exhaustively extracted two times with boiling water (200 L × 2, each extraction lasted 2 hours). The combined solutions (about 300 L) were passed over a polyamide resin column (30 L, 100–200 mesh, 20 × 200 cm) with a flow rate of 120 mL/min, and then the effluent was chromatographed on a macroporous resin column (25 L, 20–40 mesh, 20 × 200 cm) using EtOH–H_2_O (70:30, 30 L, flow rate 100 mL/min) as eluent to afford the crude saponin fraction. The 70% EtOH combined elution was concentrated *in vacuo*. The residue (a total of about 350 g) was then subjected in four portions (90 g of residue each) to MPLC with a ODS column (800 g, 25–50 μm, 6.5 × 50 cm) using a continuous gradient of MeOH–H_2_O–HCOOH (50:50:1, 55:45:1, 60:40:1, 65:35:1, *v*/*v*, each 4 L) to produce four fractions (Fr. I–IV). Fr. II (about 45 g) was further subjected to MPLC chromatography on an ODS column with a gradient of MeOH–H_2_O–HCOOH (50:50:1, 52:48:1, 54:46:1, 56:44:1, 58:42:1, 60:40:1, each 3 L, *v*/*v*) as eluent to afford six subfractions (SFr. IIa–IIe). SFr. IIa was subjected to preparative HPLC with CH_3_CN–H_2_O–HCOOH (27:73:1, flow rate 10 mL/min) elution to give compound **2** (55 mg, t_R_ 19.2 min). SFr. IIb was purified by preparative HPLC using CH_3_CN–H_2_O–HCOOH (27:73:1, flow rate 10 mL/min) to give compound **1** (32 mg, t_R_ 22.6 min) and compound **10** (72 mg, t_R_ 34.2 min). SFr. IIc was subjected to preparative HPLC with CH_3_CN–H_2_O–HCOOH (30:70:1, flow rate 10 mL/min) as eluent to afford compound **3** (38 mg, t_R_ 22.6 min), compound **4** (205 mg, t_R_ 29.1 min) and compound **8** (26 mg, t_R_ 37.5 min). SFr. IId was chromatographed by preparative HPLC and eluted with CH_3_CN–H_2_O–HCOOH (32:68:1, flow rate 10 mL/min) to give compound **5** (81 mg, t_R_ 26.1 min) and compound **7** (34 mg, t_R_ 34.5 min). SFr. IIe was subjected to preparative HPLC with CH_3_CN–H_2_O–HCOOH (35:65:1, flow rate 10 mL/min) to produce compound **6** (87 mg, t_R_ 30.1 min) and compound **9** (40 mg, t_R_ 39.4 min). Separation of fraction III (about 75 g) by MPLC with ODS column chromatography yielded **11** (6.2 g) using CH_3_CN–H_2_O–HCOOH (40:6:1, flow rate 30 mL/min) as eluent.

### 3.4. Physical Data of New Compounds

#### *Licorice-Saponin P2* (**1**)

White amorphous powder; UV (MeOH) λ_max_ (log ε) 250.2 (4.14) nm; IR (KBr) *v*_max_ (cm^−1^): 3463, 2964, 1751, 1652; ^1^H-NMR (C_5_D_5_N, 500 MHz) and ^13^C-NMR (C_5_D_5_N, 125 MHz) spectral data see Table 1; HR-TOF-MS: *m/z* 839.4120 [M+H]^+^ (calcd. for C_42_H_63_O_17_, 839.4065).

#### *Licorice-Saponin Q2* (**2**)

White amorphous powder; UV (MeOH) λ_max_ (log ε) 250.2 (4.16) nm; IR (KBr) *v*_max_ (cm^−1^): 3438, 2975, 1751, 1652; ^13^C-NMR (C_5_D_5_N, 125 MHz) spectral data see Table 1; HR-TOF-MS: *m/z* 839.4069 [M+H]^+^ (calcd for C_42_H_63_O_17_, 839.4065).

### 3.5. Acid Hydrolysis

The configuration of the sugars of compounds **1** and **3** was determined by acid hydrolysis and GC experiments based of the literature procedure [6,9]. The specific steps were as follows: a solution of compounds **1–3** (1.0 mg each) in 1 N HCl (1 mL) was stirred at 90 °C for 2 h. After cooling, the solution was evaporated under a stream of N_2_. Anhydrous pyridine solutions (0.1 mL) of each residue and l-cysteine methyl ester hydrochloride (0.06 N) were mixed and warmed at 60 °C for 1 h. The trimethylsilylation reagent trimethylsilylimidazole (0.15 mL) was added, followed by warming at 60 °C for another 30 min. After drying the solution, the residue was partitioned between H_2_O and CH_2_Cl_2_ (1 mL, 1:1 *v*/*v*). The CH_2_Cl_2_ layer was analyzed by GC/MS. The peaks of authentic sample of D-glucuronic acid after treatment in the same way were detected at 14.23 min. The final result was to compare the retention times of monosaccharide derivatives with standard sample. The absolute configuration of sugar was confirmed to be d-glucuronic acid (d-glucuronic acid for compound **1** with retention time 14.21 min; d-glucuronic acid for compound **3** with retention time 14.22 min).

### 3.6. Cell Assay

Isolated rat hepatocytes were prepared from male Wistar rats by a collagenase perfusion technique as described previously [17]. The d-GalN concentration used for cell culture treatment was previously determined according to a modification of the method of Morikawa *et al.* [18]. The cultured cells in logarithmic growth phase were made into a single-cell suspension and seeded in 96-well plates (1 × 10^4^ cells/well) in the DMEM/F 12 with 2% FBS complete medium for 24 h at 37 °C. Then, the hepatocytes were exposed to 2 mM d-GalN for 2 h to induce hepatotoxocity. The medium with silibin meglumine (as positive drug, purity 95.6%, Hunan Xieli Pharmaceutical Co., Ltd., Zhuzhou, China) and different concentrations of test compounds was mixed in cell medium (final test compounds concentration were 30 μM, 60 μM and 120 μM, respectively), and incubated for 24 h. The obtained reacted supernatant was directly used to detect ALT and AST levels. The control group was a set of cells maintained in culture medium, while the model group was a set of cells maintained in culture medium and treated only with d-GalN. All data are expressed as the mean ± SD of at least three independent experiments as indicated. The test for the paired samples was used to determine statistical difference between parameters. These differences were considered significant for *p* < 0.05 or 0.01.

### 3.7. Assay for Inhibition against PLA_2_

The PLA_2_ inhibitory assays of compounds **1–11** and the positive drug diethylenetriaminepentaacetic acid (Purchased from Aladdin, Los Angeles, CA, USA, purity > 98.0%) were carried out according to the literature [19]. First of all, each tube was added with 1 mL fresh substrate buffer solution (pH = 8.2). After that, 50 μL tested compounds at various concentrations were placed at reaction tube and blank tube, respectively. As for control tube, 50 μL deionized water was instead. Then each tube incubated at 40 °C for 10 minutes. The reaction tube and blank tube were followed by the treatment with PLA_2_ enzyme (5 μL) at the concentration of 5 μg/mL. Before put them into the incubator at the temperature of 40 °C to react 30 minutes, the content of the tube should be fully blending. The optical density value of each tube was then read in an ELISA plate reader using a wavelength of 495 nm. The IC_50_ values were calculated from concentration-response curves by means of the GraphPad Prism 5.0 Software (San Diego, CA, USA). Each experiment was repeated three times to get the mean values.

## Supplementary Materials

HRESIMS and NMR spectra of the two new compounds and the ^13^C-NMR data of compound **2** and **4** are available as Supplementary Materials. Supplementary materials can be accessed at: http://www.mdpi.com/1420-3049/20/04/6273/s1.
