# Supplementary material for: Hepatoprotective Triterpene Saponins from the Roots of Glycyrrhiza inflata
Source: Molecules. 2015 Apr 9;20(4):6273–83. doi: 10.3390/molecules20046273 (PMC6272637; doi:10.3390/molecules20046273)
Supplement: Supplementary file 1 [file molecules-20-06273-s001.pdf]

Supplementary Materials

Table S1. ^{13}C (C_5D_5N, 500 MHz) NMR spectral data of compounds **4**.

Position	4	Position	4
	δ_C mult		δ_C mult
1	40.3 CH_2	22	36.6 CH_2
2	27.3 CH_2	23	23.7 CH_3
3	90.4 CH	24	64.2 CH_2
4	45.2 qC	25	17.4 CH_3
5	56.7 CH	26	19.4 CH_3
6	19.1 CH_2	27	24.3 CH_3
7	33.9 CH_2	28	29.3 CH_3
8	46.4 qC	29	20.6 CH_3
9	62.7 CH	30	181.7 qC
10	37.8 qC	1'	105.3 CH
11	200.5 qC	2'	81.6 CH
12	129.4 CH	3'	78.6 CH
13	170.6 qC	4'	78.3 CH
14	44.4 qC	5'	74.1 CH
15	27.4 CH_2	6'	173.4 qC
16	27.3 CH_2	1''	105.5 CH
17	33.3 qC	2''	76.5 CH
18	47.5 CH	3''	78.5 CH
19	40.6 CH_2	4''	78.4 CH
20	43.4 qC	5''	73.9 CH
21	30.5 CH_2	6''	173.2 qC

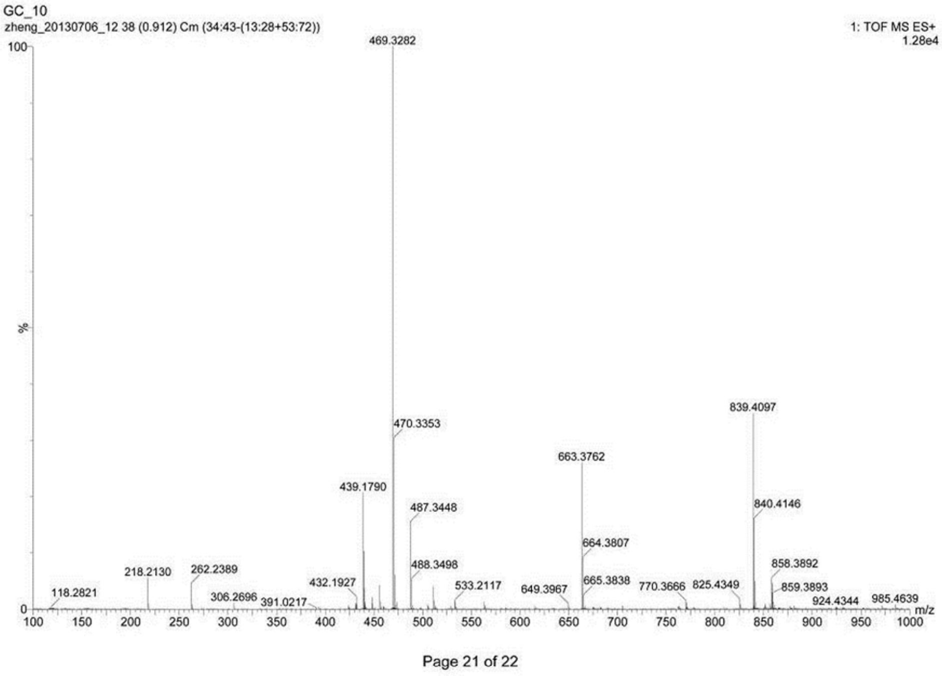

Figure S1. HRESIMS spectrum of **1**.

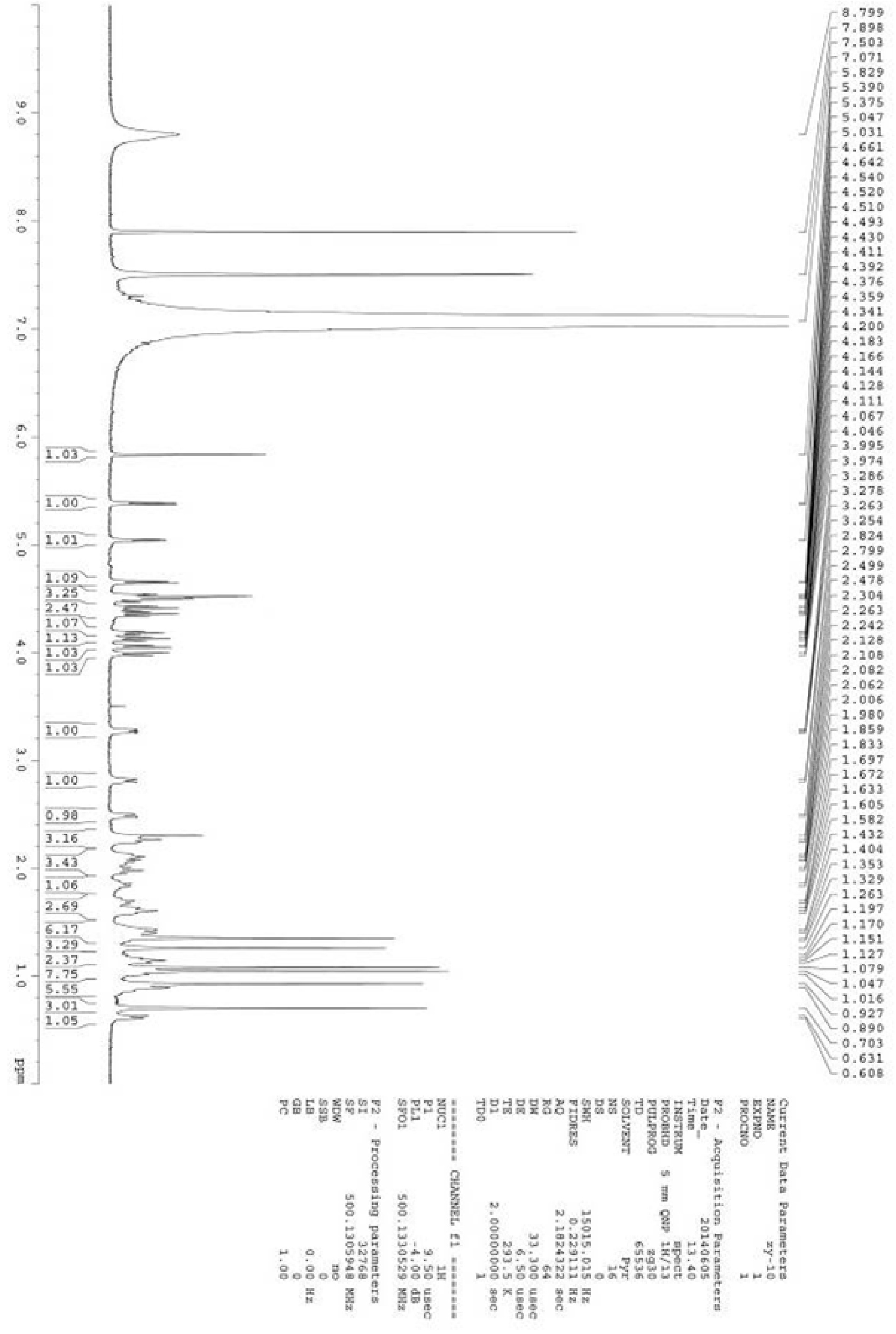

Figure S2. ^{1}H-NMR spectrum of **1** (500 MHz, C_5D_5N).

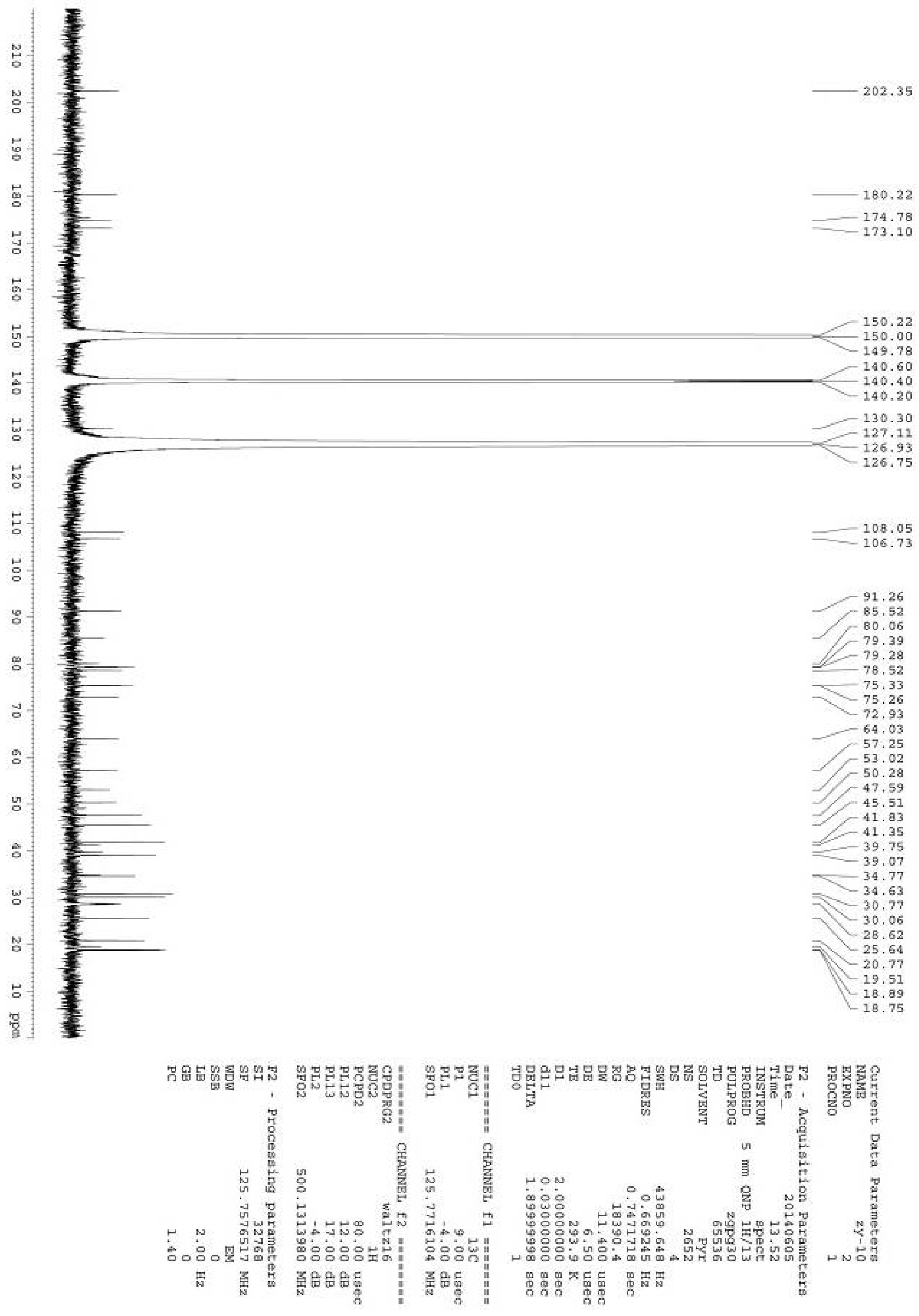

Figure S3. ^{13}C-NMR spectrum of **1** (500 MHz, C_5D_5N).

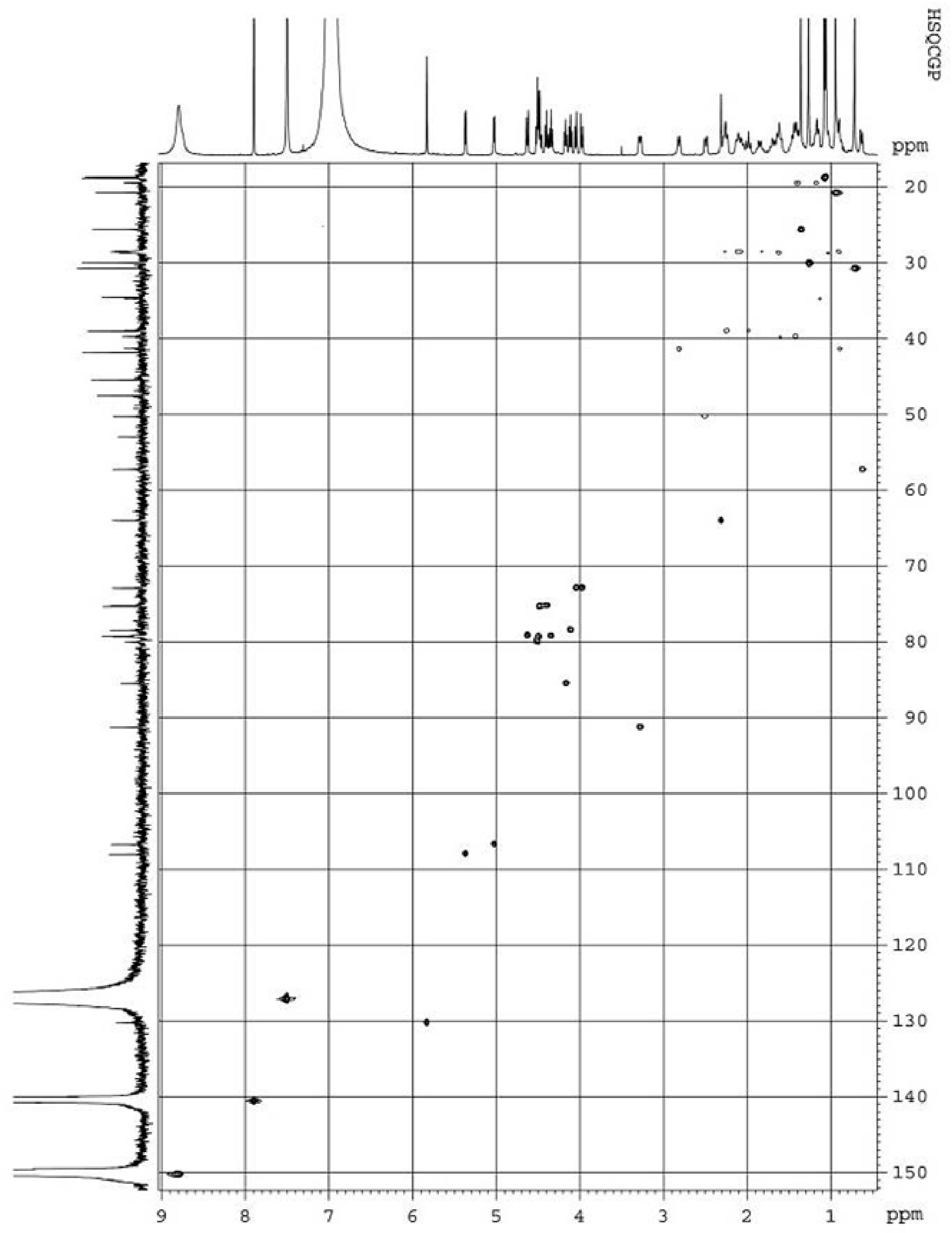

Figure S4. HSQC spectrum of **1** (500 MHz, C_5D_5N).

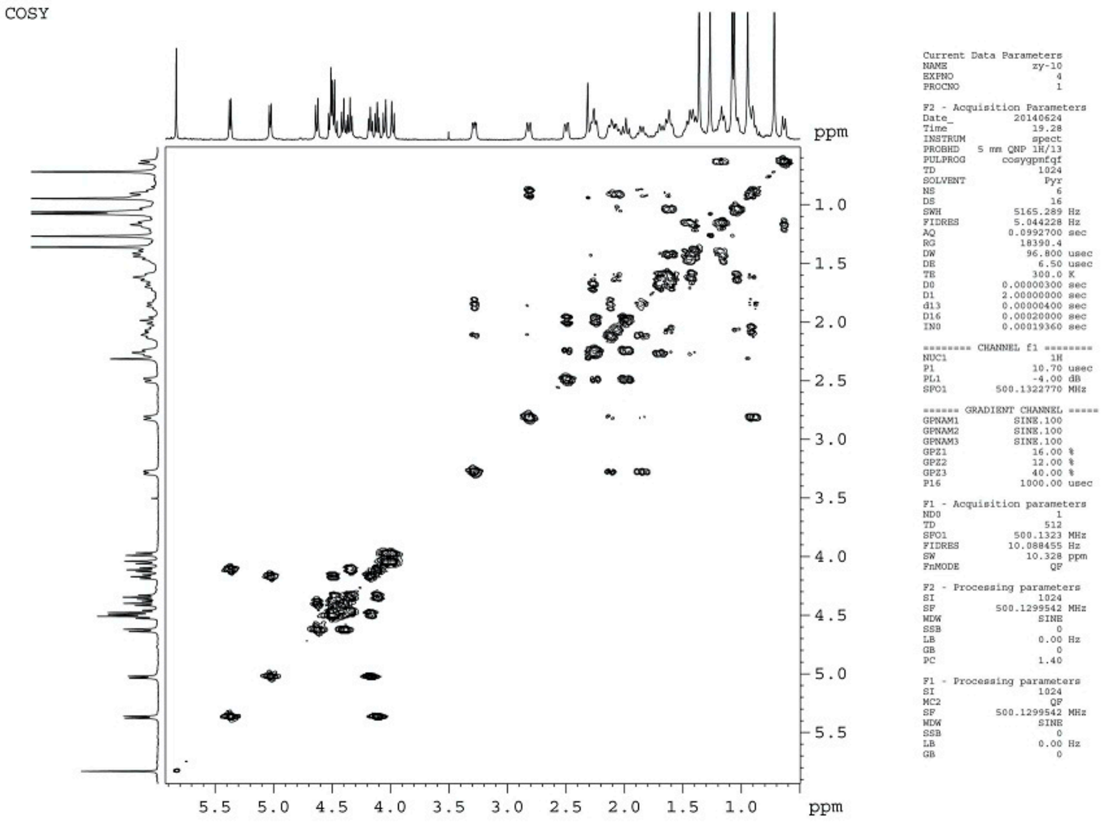

Figure S5. H-H COSY spectrum of **1** (500 MHz, C_5D_5N).

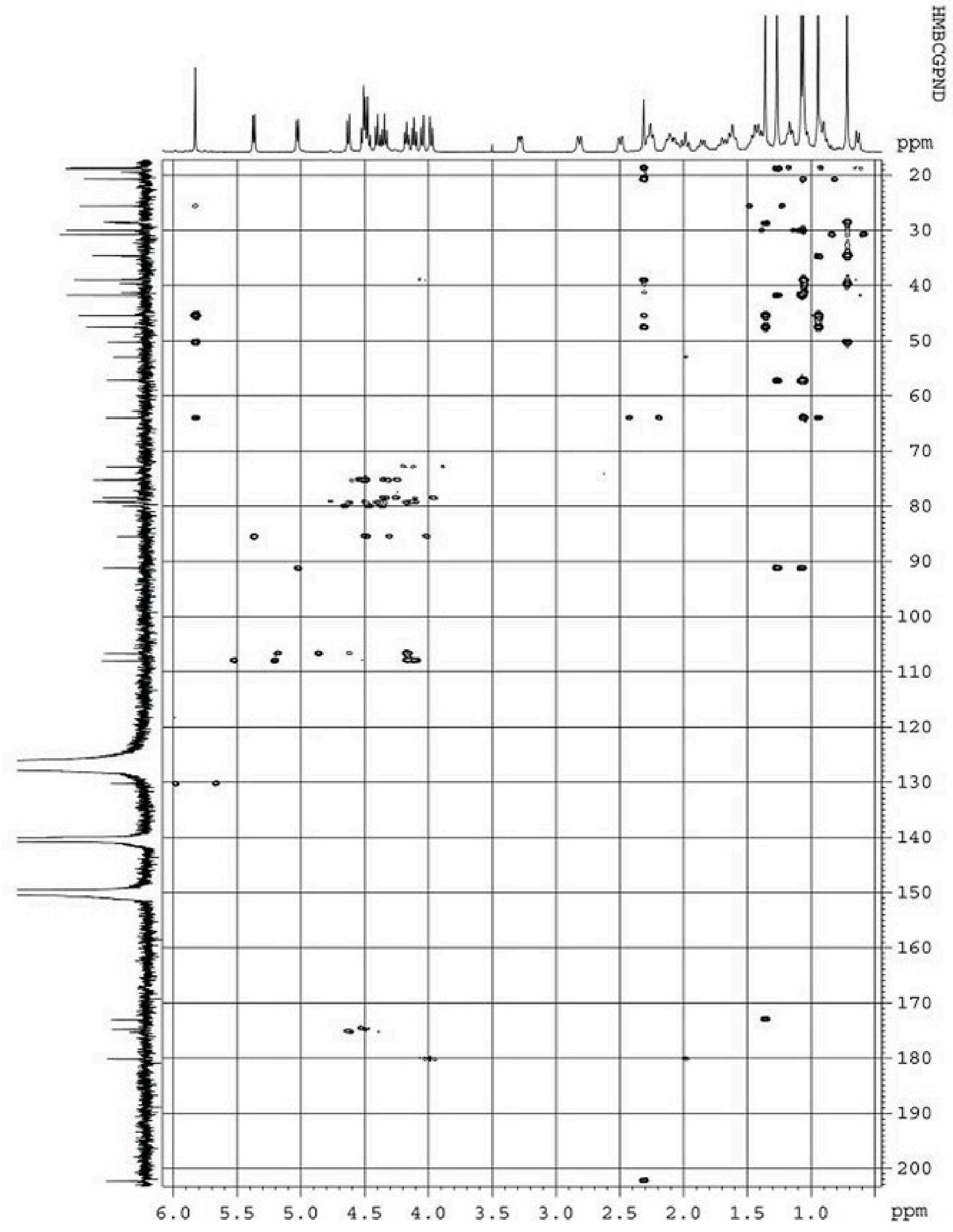

Figure S6. HMBC spectrum of **1** (500 MHz, C_5D_5N).

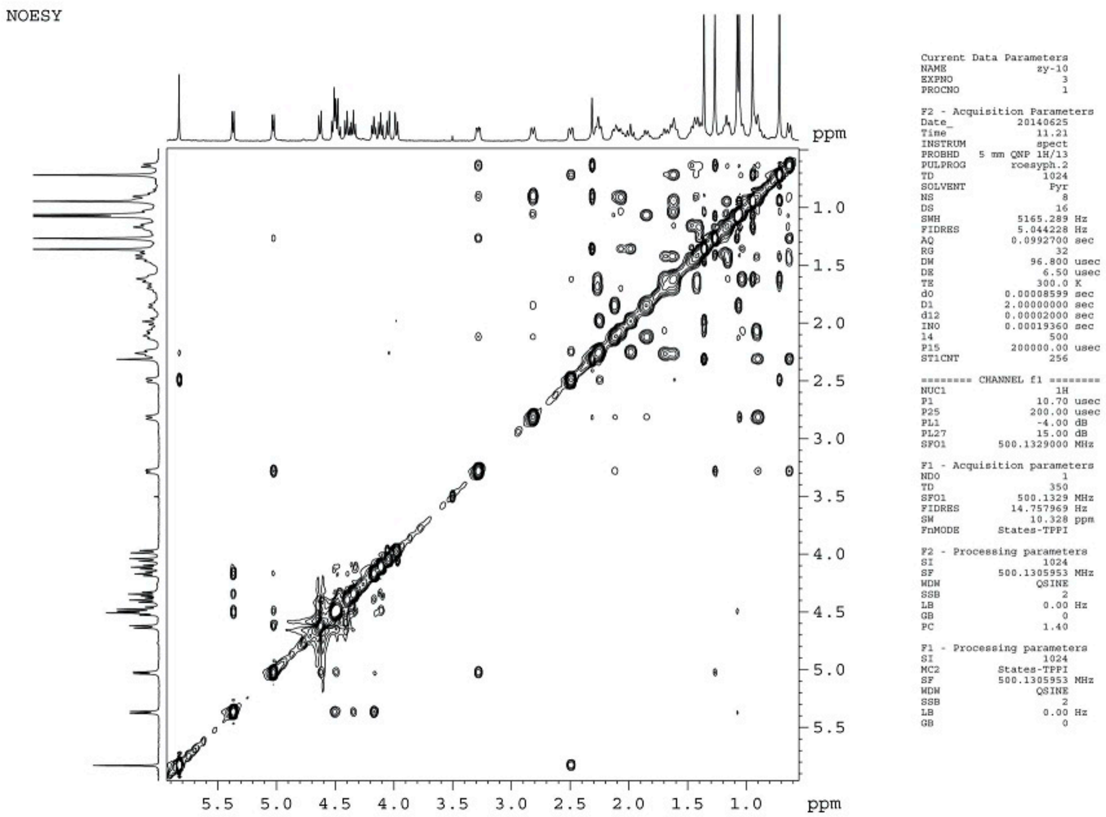

Figure S7. NOESY spectrum of **1** (500 MHz, C_5D_5N).

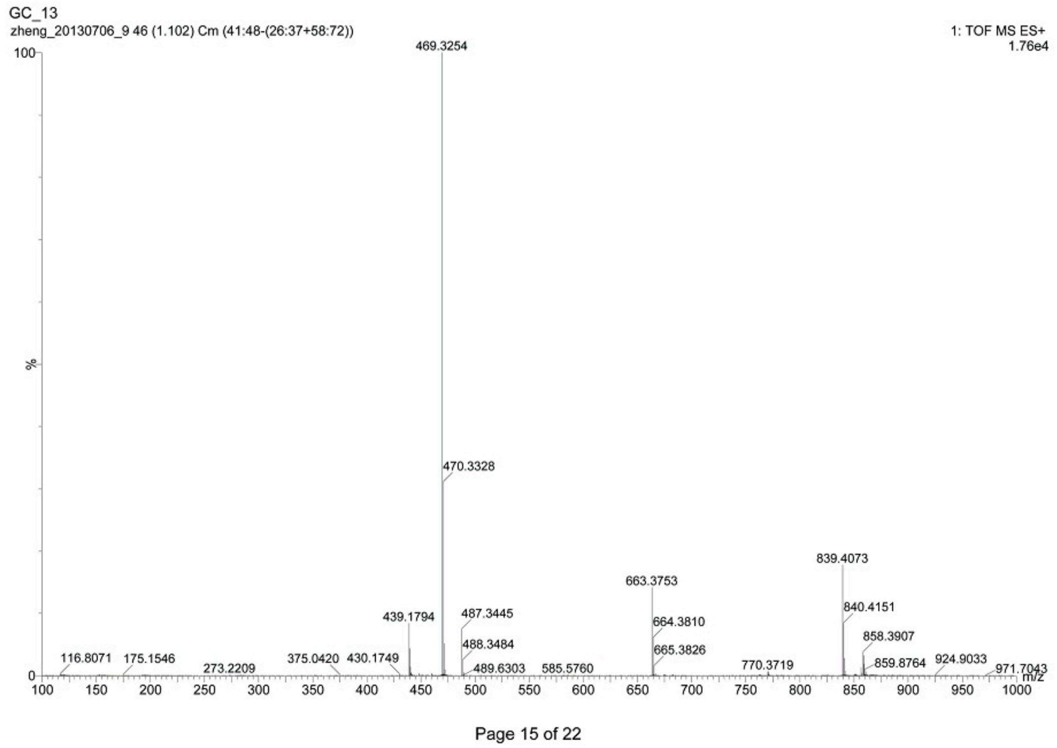

Figure S8. HRESIMS spectrum of **2**.

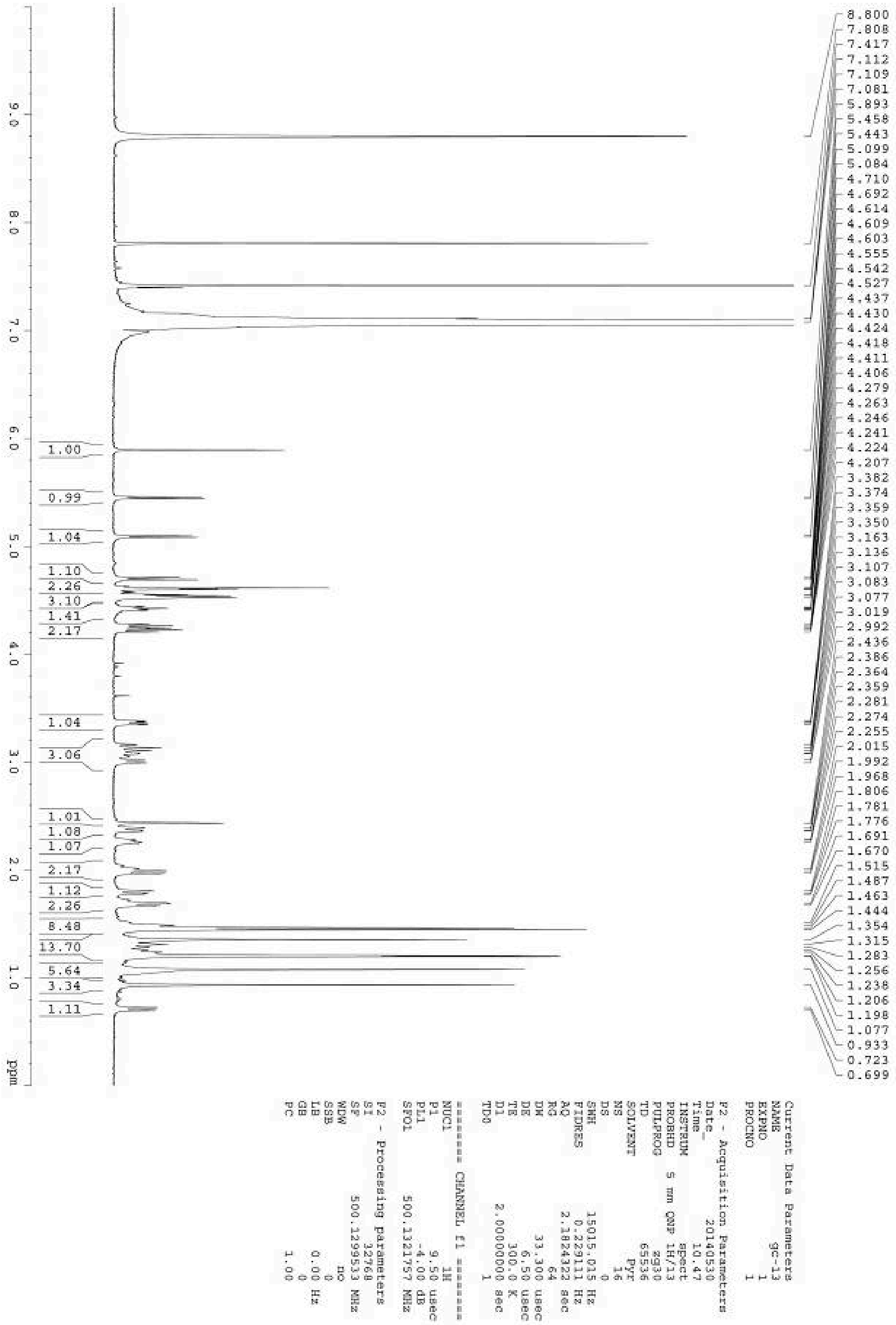

Figure S9. ^{1}H-NMR spectrum of **2** (500 MHz, C_5D_5N).

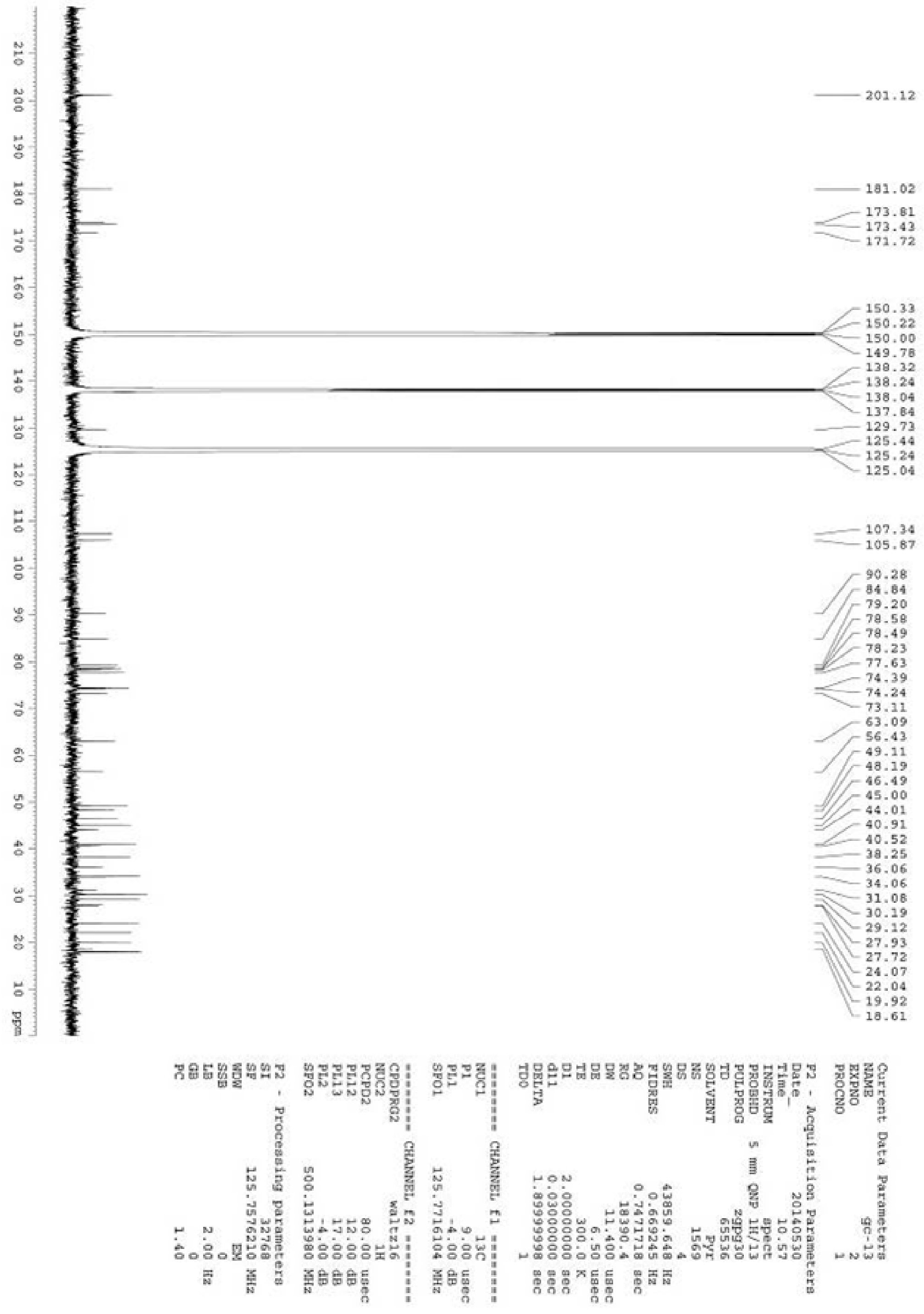

Figure S10. ^{13}C-NMR spectrum of **2** (500 MHz, C_5D_5N).

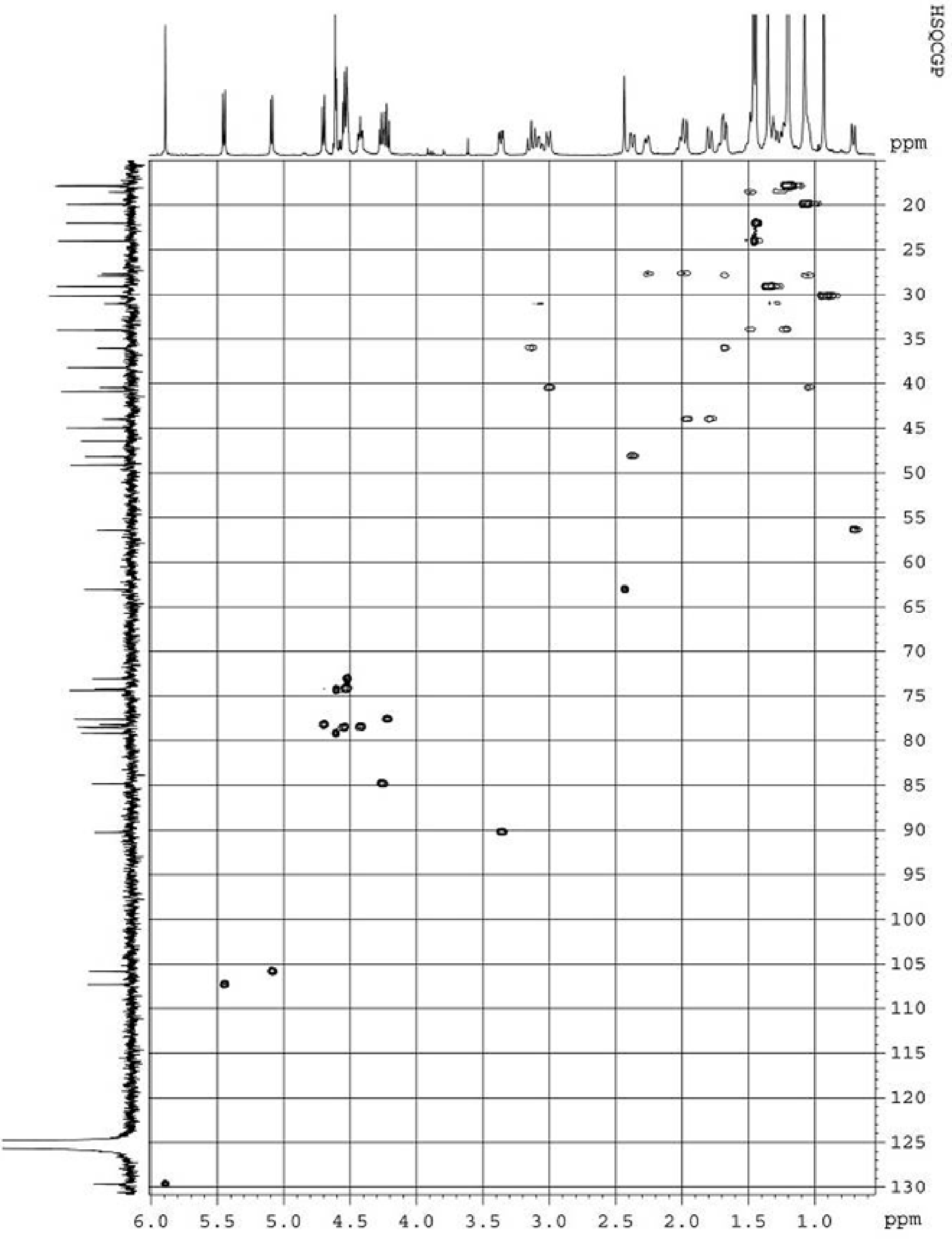

Figure S11. HSQC spectrum of **2** (500 MHz, C_5D_5N).

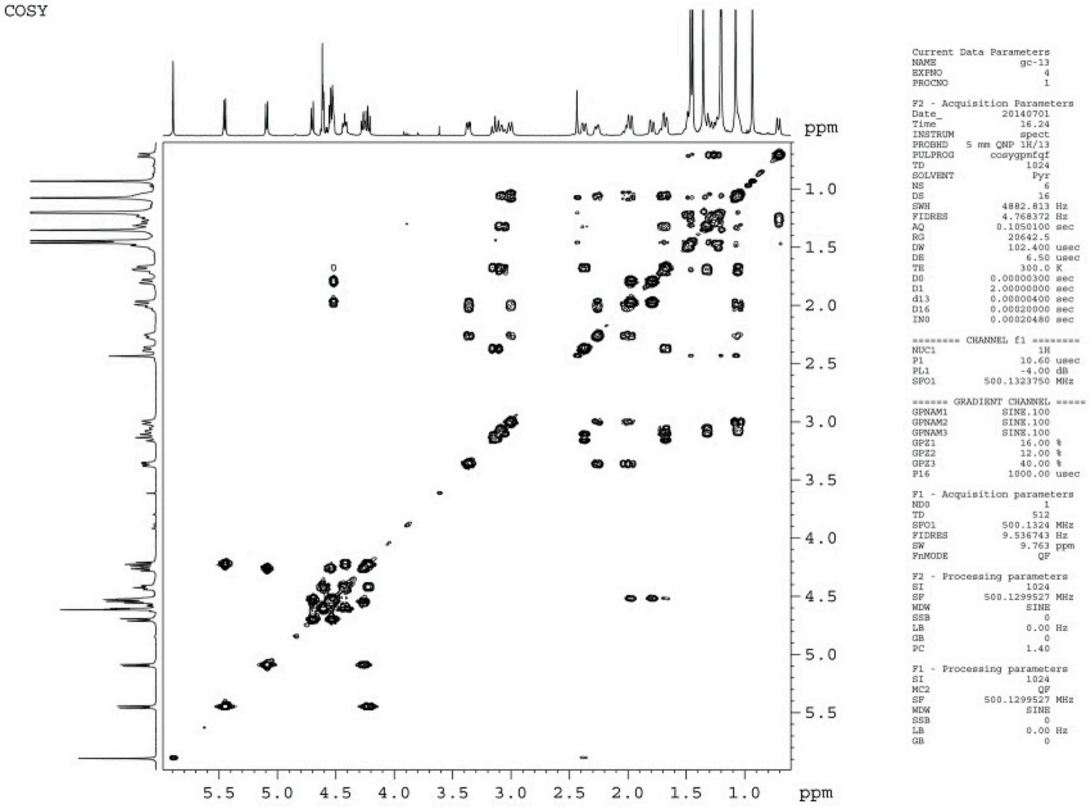

Figure S12. H-H COSY spectrum of **2** (500 MHz, C_5D_5N).

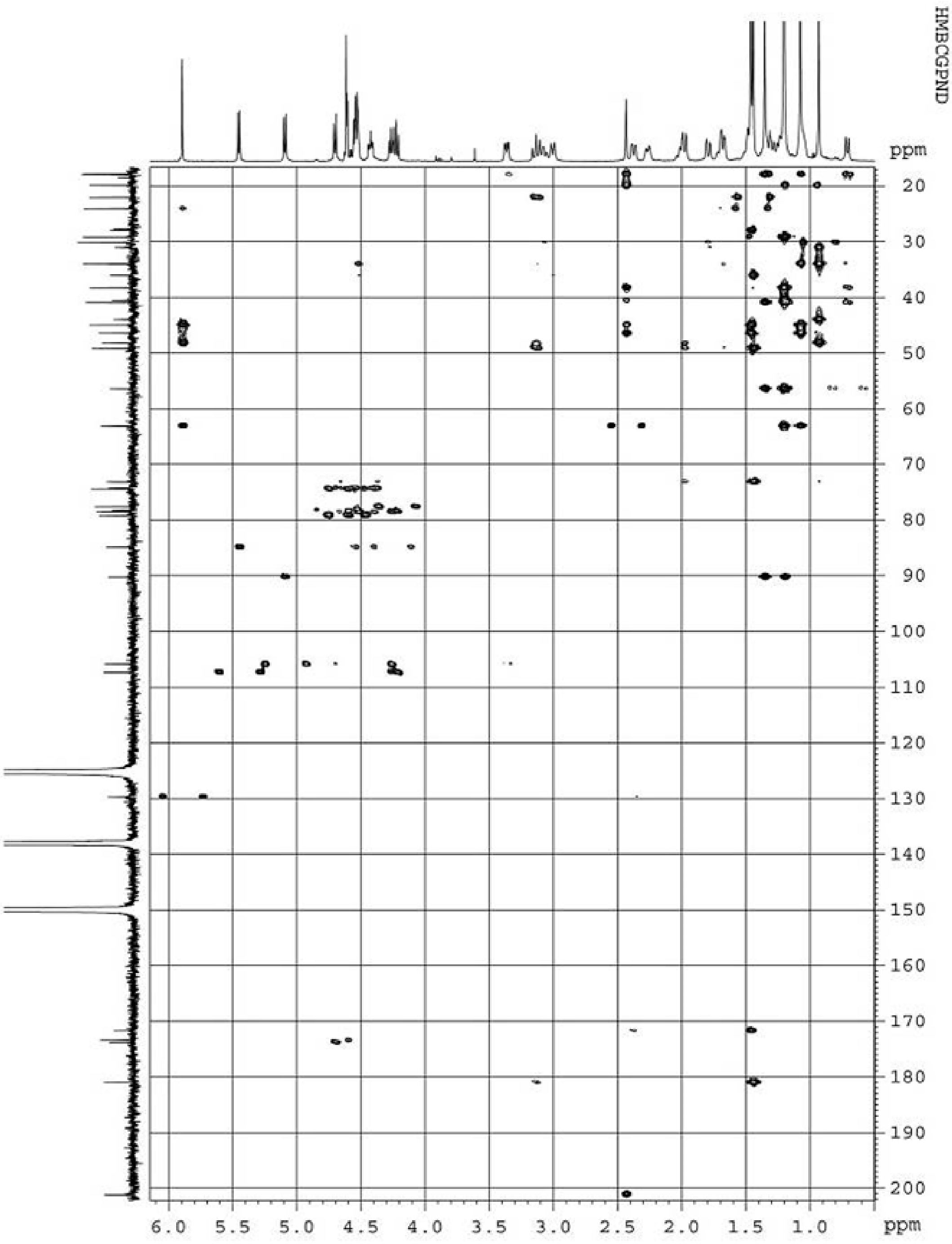

Figure S13. HMBC spectrum of **2** (500 MHz, C_5D_5N).

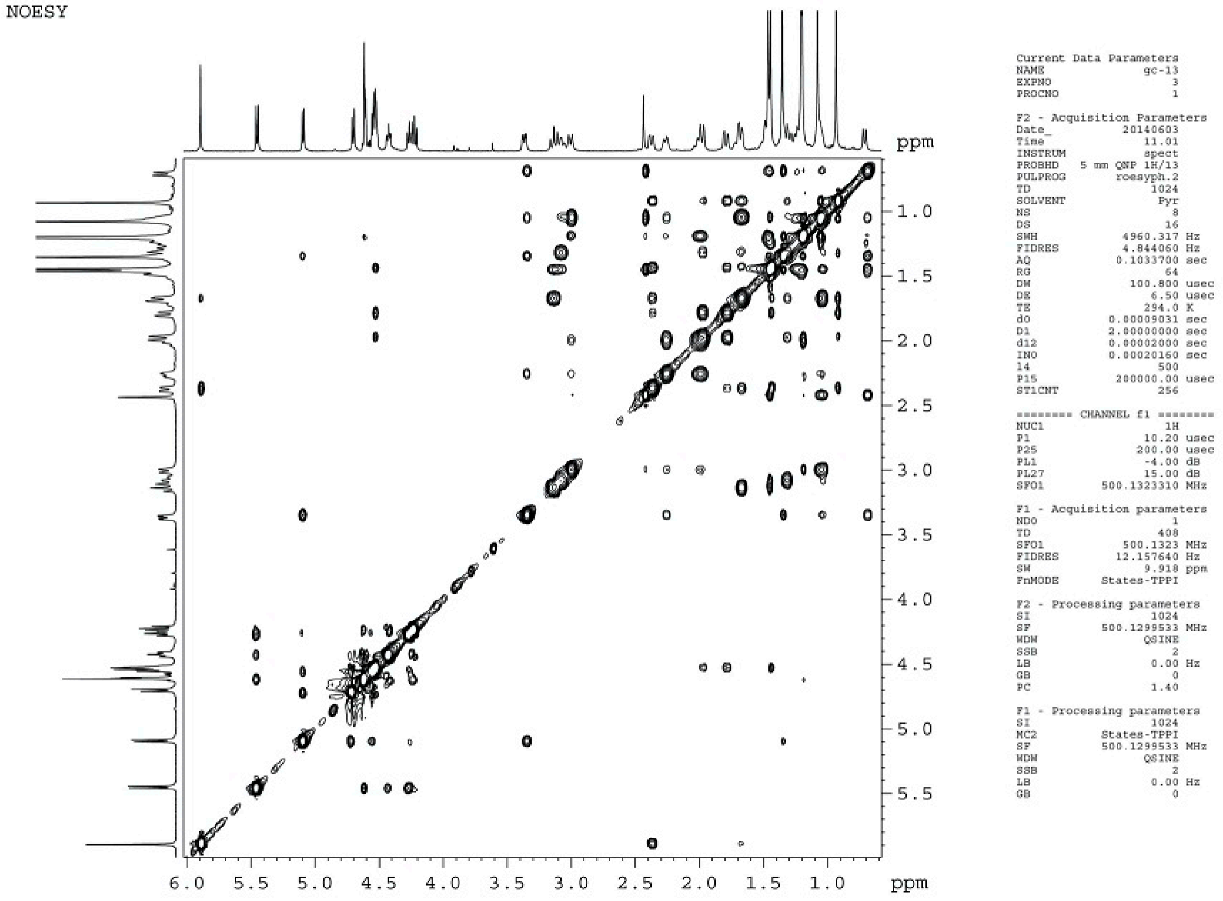

Figure S14. NOESY spectrum of **2** (500 MHz, C_5D_5N).

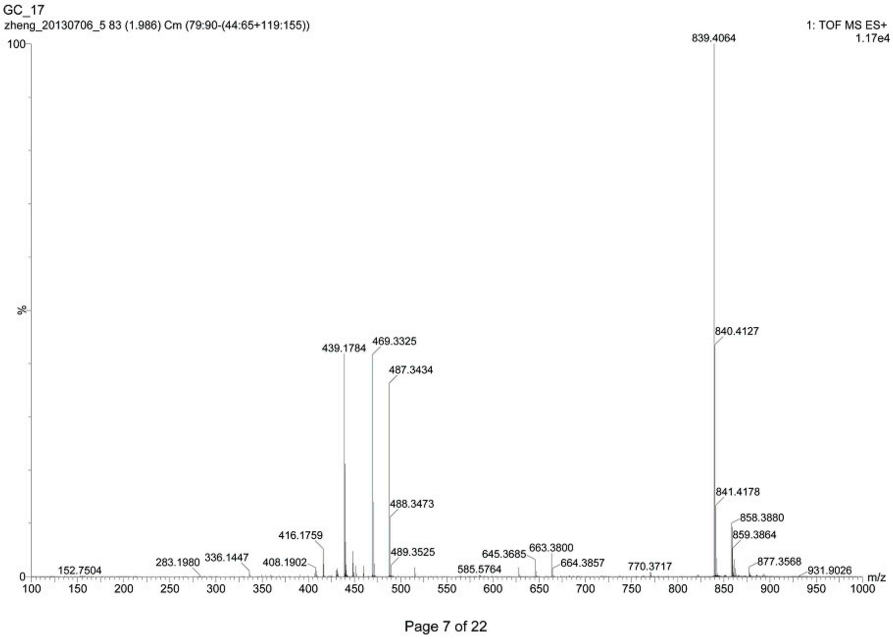

Figure S15. HRESIMS spectrum of **3**.

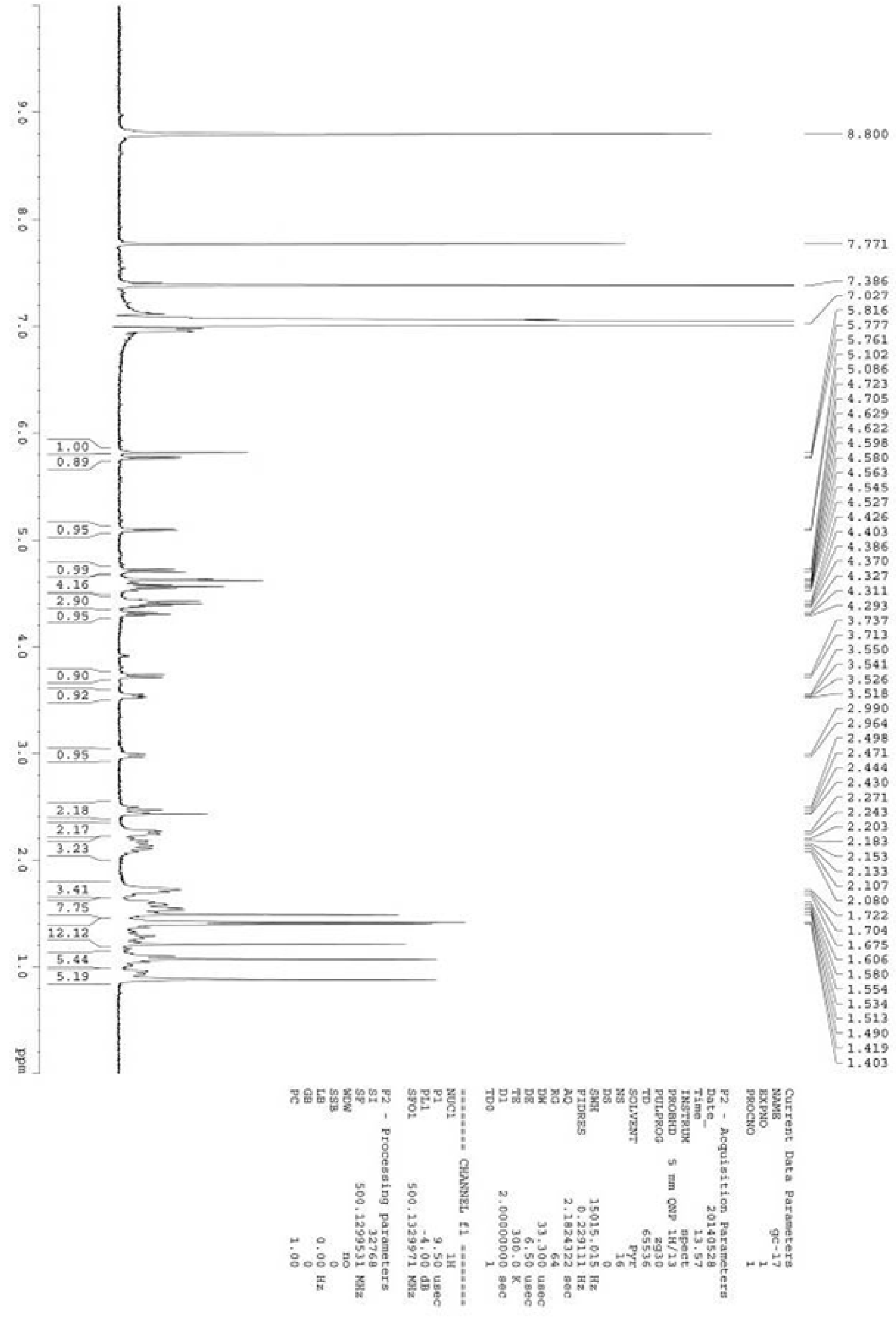

Figure S16. ^{1}H-NMR spectrum of **3** (500 MHz, C_5D_5N).

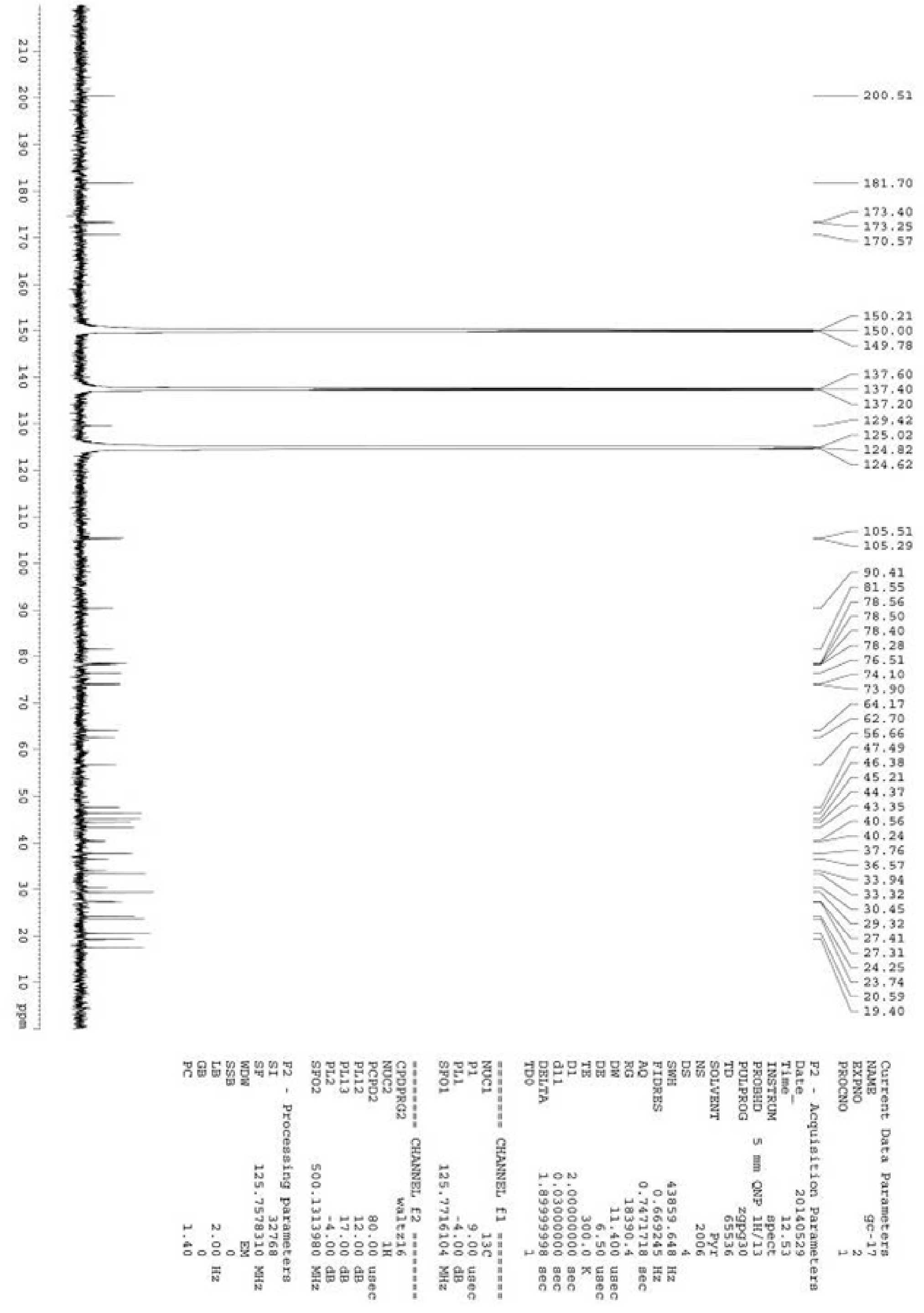

Figure S17. ^{13}C-NMR spectrum of **3** (500 MHz, C_5D_5N).

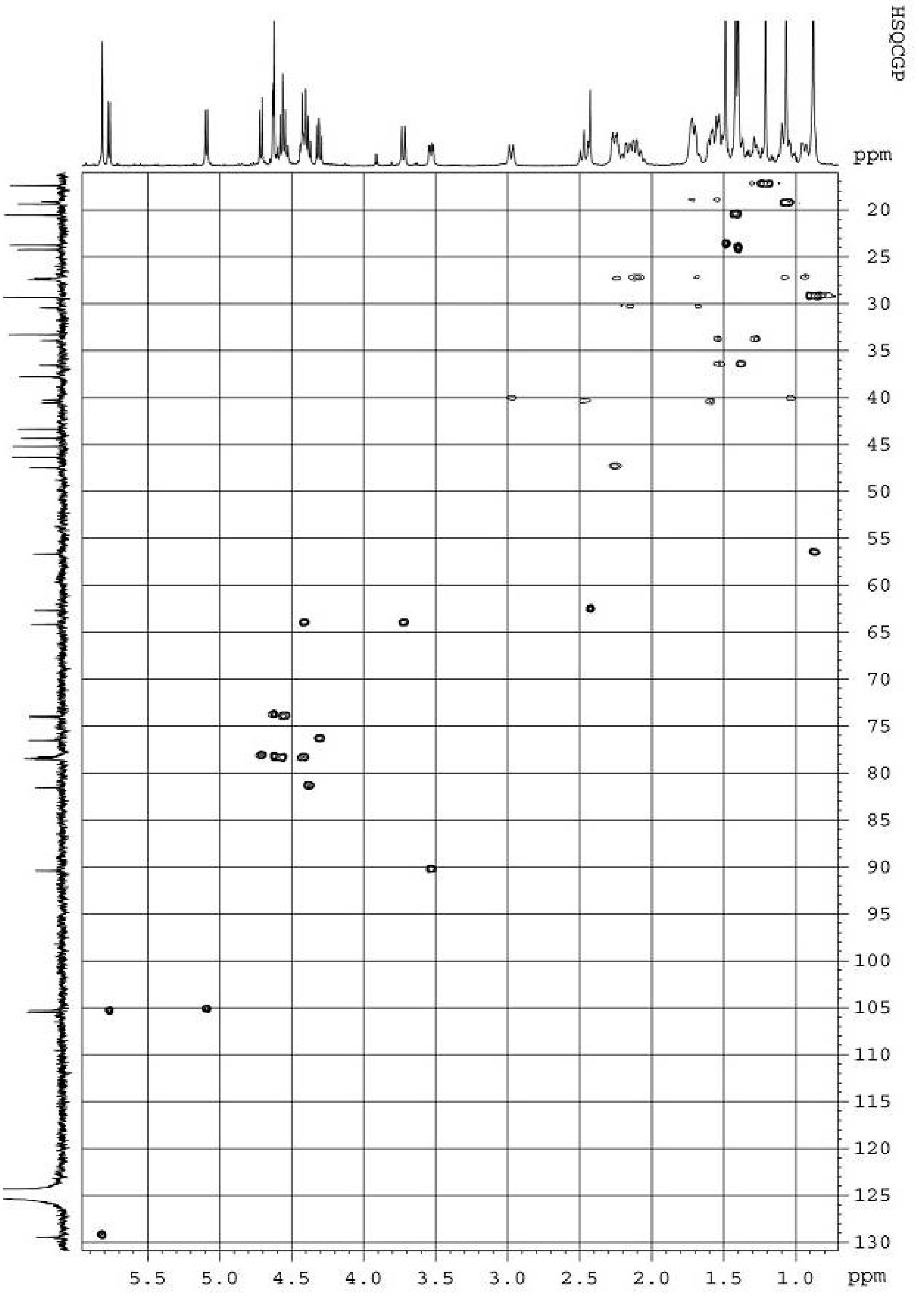

Figure S18. HSQC spectrum of **3** (500 MHz, C_5D_5N).

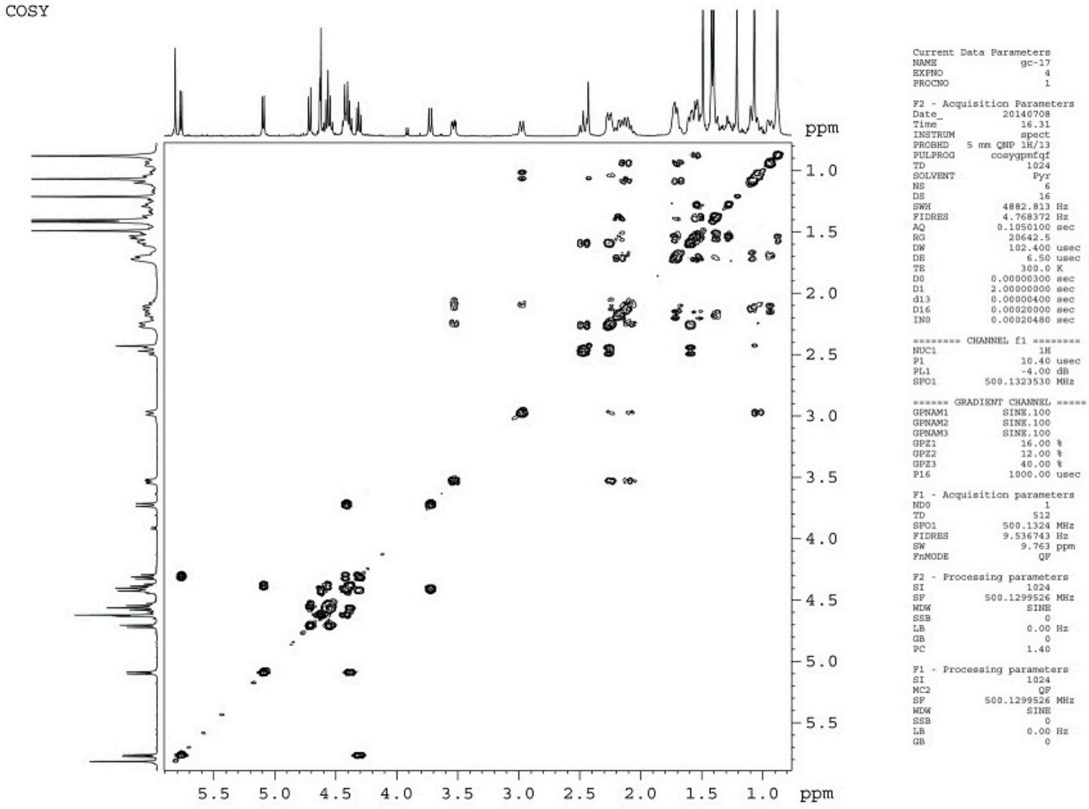

Figure S19. H-H COSY spectrum of **2** (500 MHz, C_5D_5N).

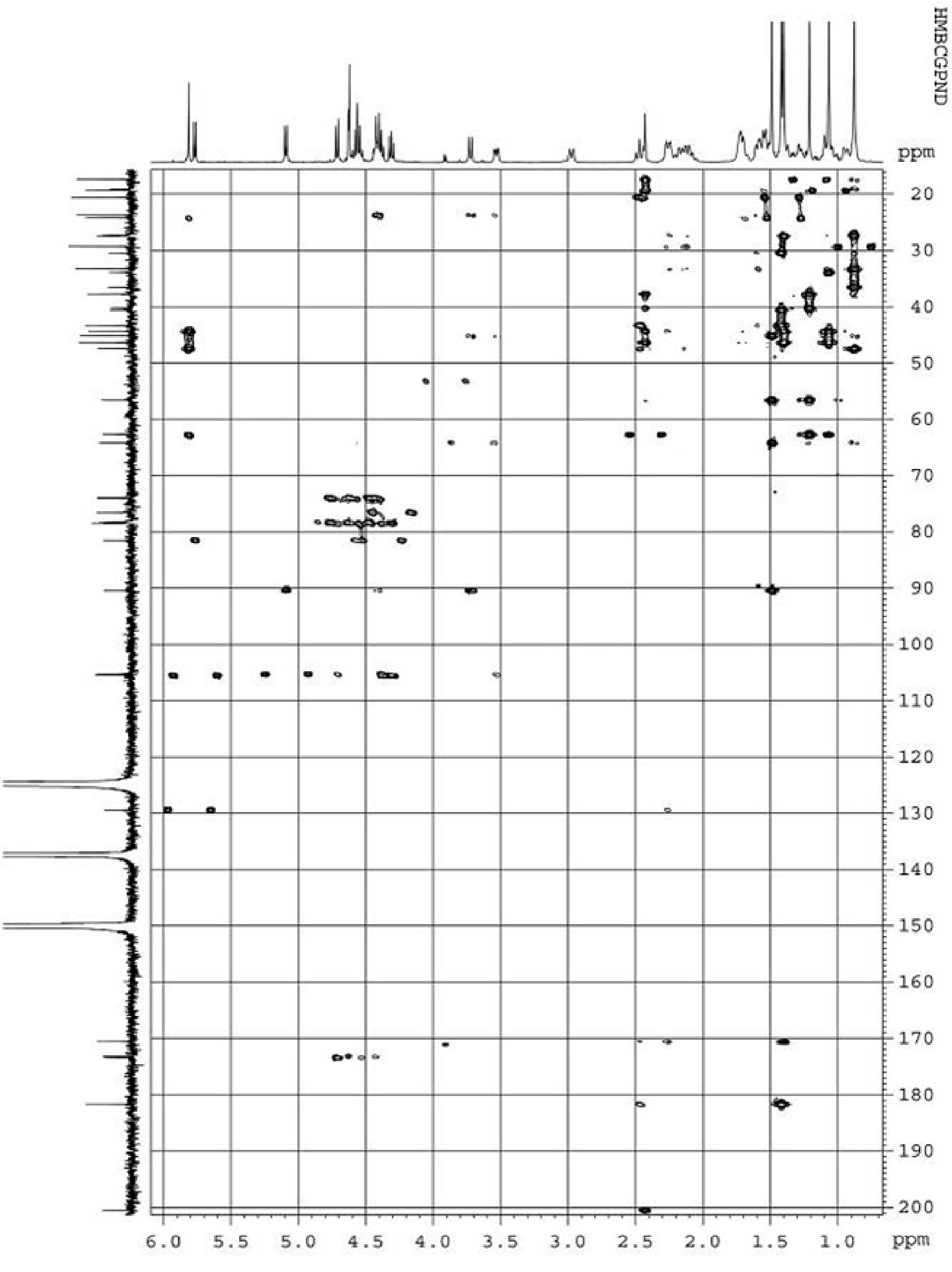

Figure S20. HMBC spectrum of **3** (500 MHz, C_5D_5N).

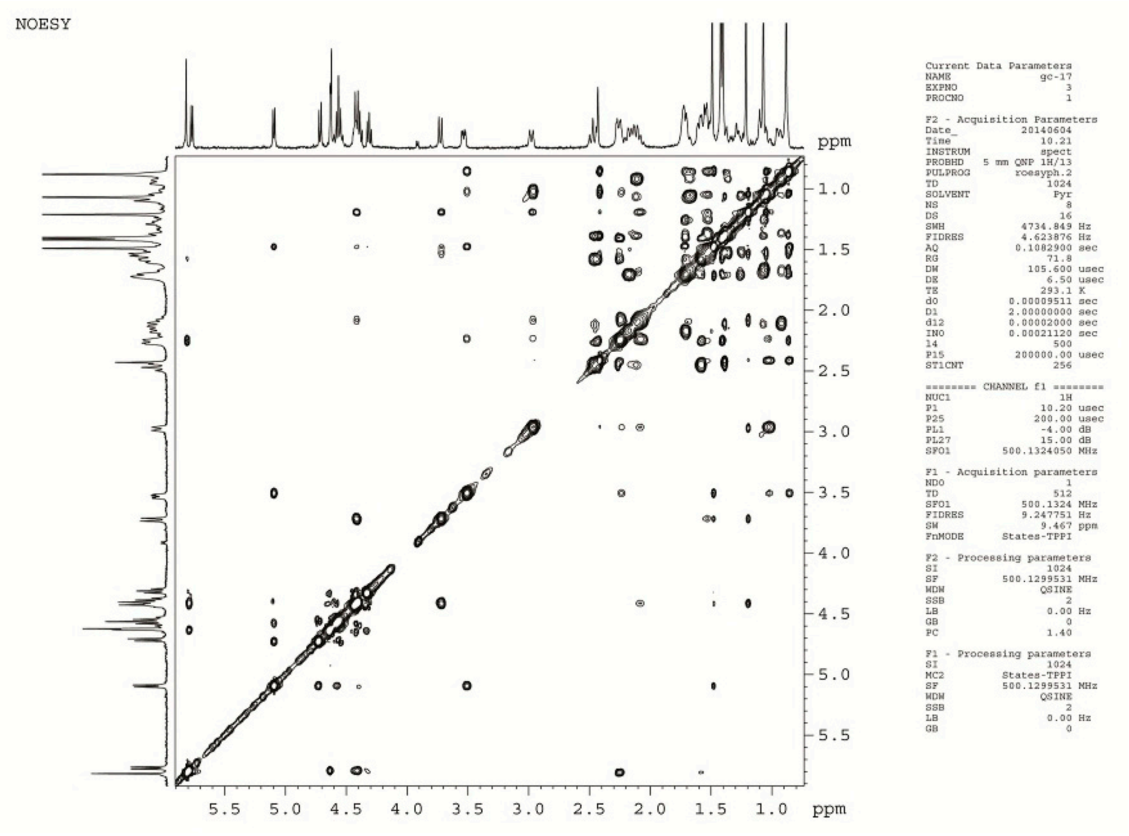

Figure S21. NOESY spectrum of **3** (500 MHz, C_5D_5N).

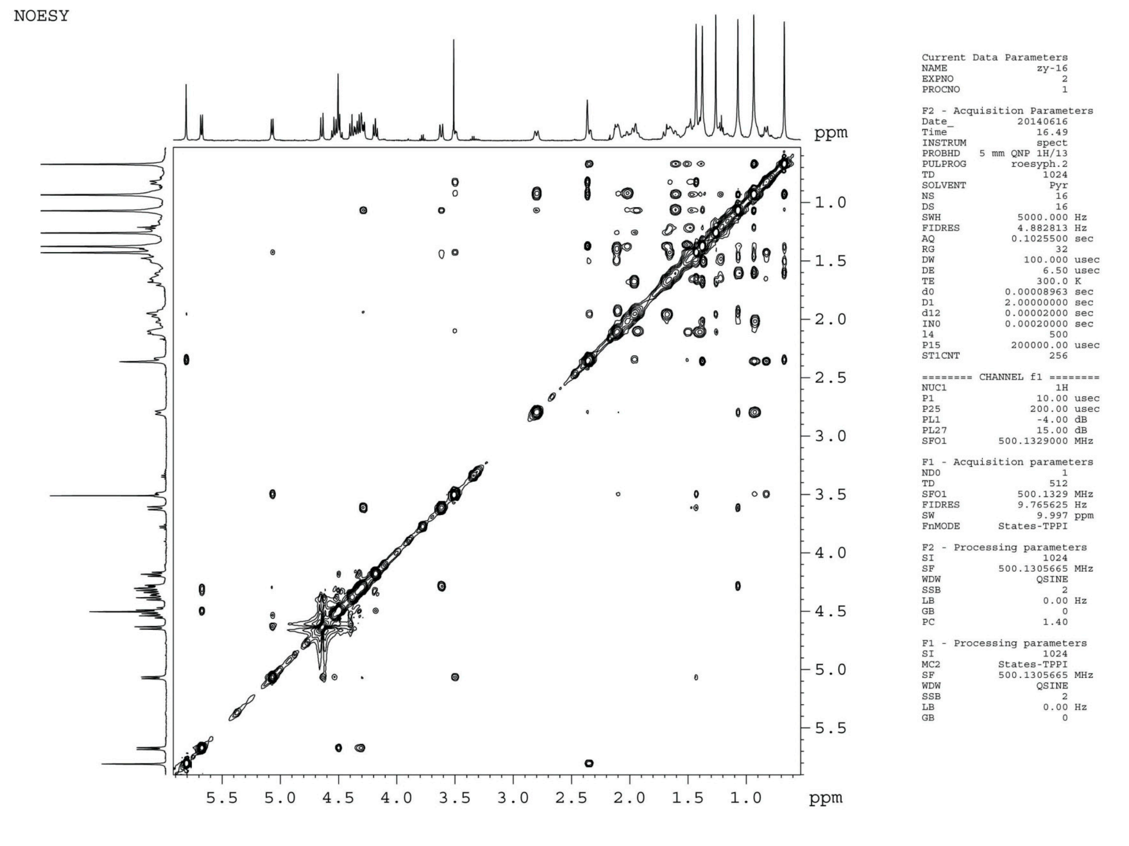

Figure S22. NOESY spectrum of **4** (500 MHz, C_5D_5N).
